# *In situ* synchrotron X-ray multimodal experiment to study polycrystal plasticity

**DOI:** 10.1107/S1600577522011705

**Published:** 2023-01-20

**Authors:** Clement Ribart, Andrew King, Wolfgang Ludwig, Joao P. C. Bertoldo, Henry Proudhon

**Affiliations:** a Mines Paris, PSL University, Centre des Matériaux (MAT), UMR7633 CNRS, 91003 Evry, France; b Synchrotron SOLEIL, L’Orme des Merisiers, BP 48, 91192 Gif-sur-Yvette, France; c Université de Lyon, INSA-Lyon MATEIS CNRS UMR 5510, 69621 Villeurbanne, France; d European Synchrotron Radiation Facility, BP 220, 38043 Grenoble, France; University of Malaga, Spain

**Keywords:** polycrystalline materials, *in situ* mechanical testing, multi-modal synchrotron experiments, diffraction contrast tomography, 3DXRD

## Abstract

The microstructure of polycrystals is known to govern the performance of structural materials. This drives the need for mechanical characterization methods capable of probing large representative volumes at the grain and sub-grain scales. In this paper, the use of *in situ* diffraction contrast tomography (DCT) along with far-field 3D X-ray diffraction (ff-3DXRD) at the Psiché beamline of Soleil is presented and applied to study crystal plasticity in commercially pure titanium. A tensile stress rig was modified to comply with the DCT acquisition geometry and used for *in situ* testing. DCT and ff-3DXRD measurements were carried out during a tensile test of a tomographic Ti specimen up to 1.1% strain. The evolution of the microstructure was analyzed in a central region of interest comprising about 2000 grains. Using the 6DTV algorithm, DCT reconstructions were successfully obtained and allowed the characterization of the evolution of lattice rotation in the entire microstructure. The results are backed up by comparisons with EBSD and DCT maps acquired at ESRF-ID11 that allowed the validation of the orientation field measurements in the bulk. Difficulties at the grain boundaries are highlighted and discussed in line with increasing plastic strain during the tensile test. Finally, a new outlook is provided on the potential of ff-3DXRD to enrich the present dataset with access to average lattice elastic strain data per grain, on the possibility of performing crystal plasticity simulations from DCT reconstructions, and ultimately on comparisons between experiments and simulations at the scale of the grain.

## Abstract 2

*In situ* diffraction contrast tomography has been deployed at the Psiché beamline of Soleil. The evolution of lattice rotation in a 2000 grain region of a titanium sample under tension is studied.

## 1. Introduction

Establishing microstructure–property relationships remains a critical engineering challenge for advanced structural materials. Metals display a heterogeneous polycrystalline organization which governs their performance and thus drives the need for mechanical characterization methods capable of probing large representative volumes at the grain and sub-grain scales. For many years, research teams have studied mechanisms closely related to these scales such as crystal plasticity, damage, fatigue or crack propagation (Pearson, 1975 ▸; Jones & Hutchinson, 1981 ▸; Roters *et al.*, 2010 ▸; Pineau *et al.*, 2016 ▸).

A large range of characterization techniques is available to probe deformation mechanisms linked to the material microstructure. Historically, investigations were either limited to surfaces (Gourgues, 2002 ▸; Wang *et al.*, 2010 ▸; Guo *et al.*, 2014 ▸; Chen & Daly, 2018 ▸; Chen *et al.*, 2018 ▸) or required destructive operations (Echlin *et al.*, 2012 ▸; Rowenhorst *et al.*, 2020 ▸).

Recent progress in synchrotron (Maire & Withers, 2014 ▸; Nygren *et al.*, 2020*a* ▸) and laboratory X-ray techniques (Bachmann *et al.*, 2019 ▸) has paved the way to a paradigm shift sometimes called ‘diffraction microstructure imaging’ (DMI), leading to increasingly complex multimodal *in situ* experiments that allow observations to be made non-destructively and concurrently on several scales, resulting in significantly richer datasets. In particular, diffraction contrast tomography (DCT), a near-field variant of 3DXRD (Poulsen *et al.*, 2001 ▸), allows reconstruction of mesoscopic digital grain maps (on the order of 1 mm^3^) on which simulations can be computed directly (Proudhon *et al.*, 2016 ▸; Shade *et al.*, 2019 ▸).

The convergence of experimental and numerical data leads to unified but massive datasets (Sangid, 2020 ▸); yet this wealth of information can render manual post-processing untractable. Moreover, modalities are often acquired independently which further hinders analysis. Note that efforts in the scanning electron microscope (SEM) community, also driven by equipment manufacturers, have led to successful correlative experiments using microstructural information in 3D using serial sectioning methods (Burnett *et al.*, 2014 ▸; Charpagne *et al.*, 2021 ▸).

Several recent studies took advantage of DMI for the study of polycrystal plasticity, either with single techniques (Miller & Dawson, 2014 ▸; Pagan *et al.*, 2017 ▸; Hektor *et al.*, 2019 ▸) or relying on a multimodal approach while focusing on a few grains with manual (Proudhon *et al.*, 2018 ▸; Sangid *et al.*, 2020 ▸; Nygren *et al.*, 2019 ▸, 2020*b* ▸) or statistical analysis (Nervo *et al.*, 2014 ▸; Wang *et al.*, 2021 ▸). These remain difficult studies, limited in several aspects by access to one of the few synchrotron beamlines where DMI is possible, the limited number of samples one can test during beam time, and most notably by the time needed to analyze the data produced. Regarding the latter, the absence of a stable multimodal framework in the community clearly limits the diffusion and use of these promising techniques.

On the other hand, continuous technological progress is expected to overcome these obstacles. Specifically, the recent ESRF EBS upgrade represents a significant leap with two orders of magnitude improvement in brightness, signal focusing, and spatial and time resolution (Cho, 2020 ▸). This leads the way for unmatched *in situ* testing opportunities. For instance, the duration of a single DCT scan has dramatically reduced from 1 h to 3 min. Other synchrotrons such as SOLEIL have already scheduled similar upgrades for the coming years.

As part of an effort to promote these developments, the present paper aims to introduce the deployment of an *in situ* (or 4D) X-ray multimodal technique involving DCT for users on the Psiché beamline at Synchrotron SOLEIL. In addition, the ability to acquire and reconstruct 3D polycrystalline grain maps of initial and deformed microstructures with up to 10^3^ grains is demonstrated in hexagonal titanium, allowing access to lattice curvature evolution over large datasets in the bulk of the material.

## 2. Materials

A commercially pure α-phase grade 2 titanium (CP-Ti) was used for this study (Barkia *et al.*, 2015 ▸; Marchenko *et al.*, 2016 ▸). It has a hexagonal close-packed structure with a *c*/*a* ratio of 1.586. The material was obtained from TIMET in the form of a 1.6 mm-thick rolled sheet with an initial average grain size of 15 µm and typical texture of cold-rolled Ti (Keeler & Geisler, 1956 ▸). The chemical composition of the batch was reported as follows (in wt%): 0.14 Fe, 0.005 C, 0.08 O, 0.008 N.

Prior to sample preparation, a heat treatment was applied in order to increase the grain size to 50 µm over 24 h (below the β-transus at 855°C) with 10 l min^−1^ argon flux and followed by air quenching.

Samples were machined by electron discharge machining (EDM) along the rolling direction (RD) with a dog-bone shape (20 mm long with a 5 mm gauge length) and an initial 600 µm square cross section. In this case a volume of 1 mm height contains around 10 000 grains. Circular pin holes of 1 mm diameter were drilled symmetrically in each of the specimen heads to apply the mechanical load. All four sample faces were then pre-polished by mechanical grinding with a 1200 grit sandpaper. One face was further polished with EBSD (electron back-scattered diffraction) quality by an additional 2400 and 4000 grit pre-polishing followed by a 25 h vibratory OPS cycle using a QATM Qpol Vibro polisher and 50% Eposil M/50% distilled water solution. Eventually, fiducial micro-indents were added close to the center of this face [shown in Fig. 4(*a*)] to define a region of interest (ROI) for the present study. Reference SEM imaging covering the central zone delimited by the indents was then obtained by a mosaic of secondary electron (SE) images and EBSD. Prior to the synchrotron *in situ* experiment, reference tensile curves up to 3% total strain were obtained via laboratory tensile tests. After the *in situ* test, new reference acquisitions were performed for validation of the Psiché DCT data. The ROI of the sample was scanned again with DCT at the ID11 beamline of the ESRF using a limited aperture to reduce diffraction spot overlap. *Post mortem* EBSD was also carried out (after re-polishing the front face of the sample over a depth of 40 µm).

## 3. *In situ* experimental testing

**Figure 1:**
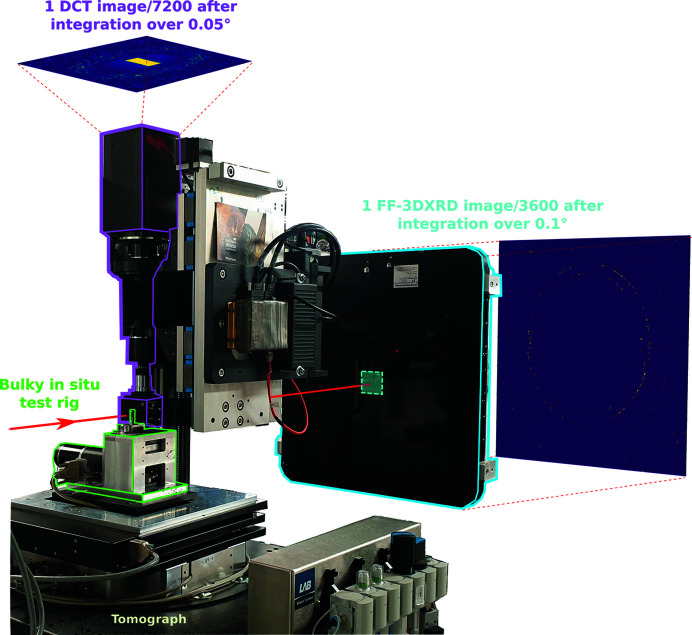
Overview of the multimodal experimental setup on the Psiché beamline at Soleil.

Experiments were carried out on the Psiché beamline to implement the *in situ* multimodal acquisition with DCT, far-field 3DXRD (ff-3DXRD) and phase contrast tomography (PCT), each technique providing complementary information on the deformation event during the mechanical test. The beamline was configured with a 40 keV monochromatic beam. An overview of the multimodal experimental setup is shown in Fig. 1 ▸.

The near-field detection system used for PCT and DCT acquisitions is composed of a 0.8 mm × 1 mm tungsten beamstop, a 50 µm-thick LuAG scintillator, a 45° angle deflecting mirror and a 4608 × 2592 pixel Hamamatsu ORCA-Lightning Digital CMOS camera mounted with a 5× optical magnification lens giving an effective pixel size set to 1.087 µm.

The far-field acquisition system is made of an attenuation block composed of two 10 mm-thick glass slabs to avoid saturating the detector, a 2048 × 2048 pixel Perkin-Elmer XRD 1621 CN3 X-ray detector with a 200 µm pixel size and a lead beam stop directly mounted on the detector screen. Note that in the present work the DCT and ff-3DXRD scans were carried out sequentially but in principle could be performed simultaneously using a semi-transparent mirror.

**Figure 2:**
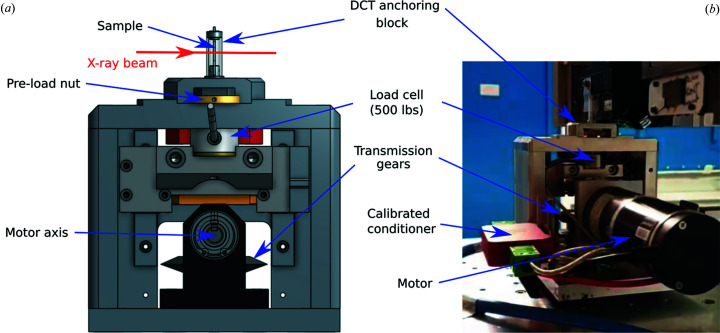
*In situ* test rig Bulky for DCT acquisition. (*a*) 3D sketch front overview of Bulky with the modified grip system compatible with DCT. (*b*) Bulky installed on Psiché tomograph in the DCT acquisition configuration.

The *in situ* test rig Bulky, designed at the Centre des Matériaux, was used for the experiment [see Fig. 2 ▸(*a*) and the work by Pelerin *et al.* (2019 ▸) for more details on the stress rig]. The anchoring system has been modified by integrating a quartz tube (8 mm in diameter and 1 mm thick) in order to comply with the DCT geometry, by allowing the detector to be brought as close as 5 mm to the rotation axis [Fig. 2 ▸(*b*)]. The tensile setup was calibrated with a 500 lb load cell purchased from Futek.

Bulky was installed on the tomograph of the experimental hutch of the Psiché beamline and the sample was carefully mounted to avoid causing deformation prior to loading. Scans were performed in the undeformed configuration and after two different loading steps (0.7% and 1.1% total strain) in the selected ROI. At each step, a large-field-of-view PCT scan was performed first (2 mm in height), with the detector positioned 30 mm behind the sample while removing the beamstop and switching the camera to high dynamic range mode. The sample was rotated over 360° around the tomograph vertical axis to acquire 1000 images. The initial PCT acquisition allowed the generation of a high-definition absorption contrast tomographic volume which can be used as a mask for the DCT reconstruction. In addition, the fiducial markers were visible in the reconstructed volume and were used to position the sample accurately to ensure that the same zone was illuminated at each step. Moreover, the distance between markers in the intermediate PCT reconstructions provided a direct measurement of macroscopic strain.

At each loading step, following the PCT, the detector was moved 8.5 mm behind the sample and centered in order to align the beam stop with the X-ray beam. The camera was switched to low dynamic range, the rotation speed was set to 0.05° s^−1^ and 1 s exposure time was used for each radiograph. Two 1060 µm × 278 µm box beam aperture DCT scans were performed with 50 µm overlap, resulting in a total acquired height of 470 µm. A total of 7200 diffraction images per scan over 360° were collected with an integration step of 0.1°, taking 2 h and producing 150 Gb of raw data.

**Figure 3:**
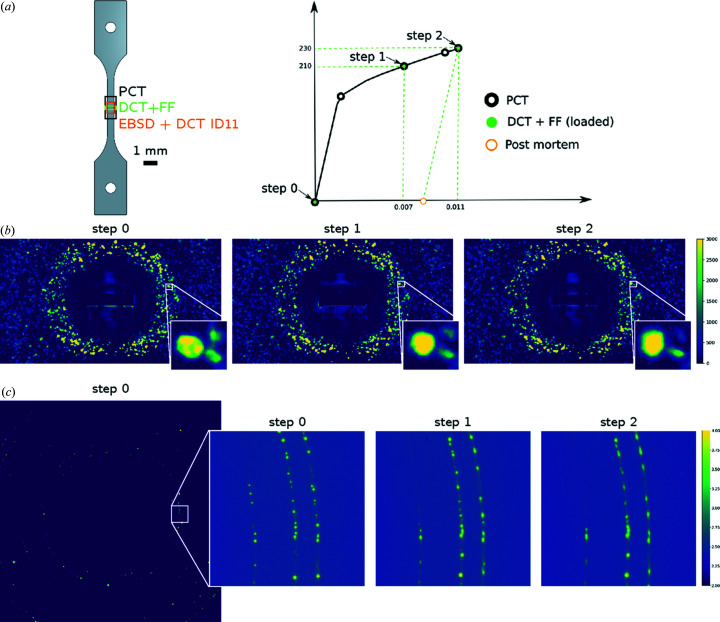
*In situ* experimental overview: (*a*) specimen geometry showing the different acquisition zones and loading curve, (*b*) near-field images integrated over 5° showing the diffraction spots acquired during DCT scans at each step (the insets show a zoomed-in view of a given location), (*c*) ff-3DXRD single frame image and zoom on a given detector zone showing 1° integrated images at each load step (logarithmic scale has been applied).

The far-field detector assembly was placed 1 m downstream of the sample, the near-field detector was moved aside and ff-3DXRD scans could be performed with the same illumination as DCT; 3600 diffraction images were taken over 360° with an integration step of 0.05°. Each scan took 15 min and represents 60 Gb of data. The complete experimental procedure is summarized in Fig. 3 ▸.

## 4. Volume reconstruction

### 4.1. PCT reconstruction

The PCT acquisition scans were reconstructed with a filtered back-projection algorithm using *PyHST2* (Mirone *et al.*, 2014 ▸). A Paganin filter (Paganin *et al.*, 2002 ▸) was also used to enhance contrast in the reconstruction. Fiducial indents are easily detected in the PCT reconstruction which allows the strain to be measured directly at each step.

### 4.2. DCT reconstruction at Psiché

Currently, DCT reconstructions are tightly linked to the ESRF computing infrastructure, using the Matlab code developed by the team working at the Materials Science beamline ID11 [https://gitlab.esrf.fr/graintracking/dct (Ludwig *et al.*, 2009 ▸; Reischig *et al.*, 2013 ▸; Viganò *et al.*, 2014 ▸)]. The key reconstruction steps consist of background correction and normalization of the collected images, diffraction spot segmentation, spot-pair matching, grain indexing, and grain-by-grain tomographic reconstruction. Microstructures exhibiting negligible intragranular orientation spread can be reconstructed using the 3D-DCT (single orientation) approach which is based on the algebraic reconstruction algorithm [simultaneous iterative reconstruction technique (SIRT)], implemented in the *ASTRA* open source tomography library (van Aarle *et al.*, 2016 ▸; Palenstijn *et al.*, 2011 ▸). For materials with non-negligible orientation spread within the grains, the 6D-DCT approach can be used in order to capture the intra-granular orientation field. This approach is based on an in-house implementation of the Chambolle–Pock optimization algorithm (Chambolle & Pock, 2016 ▸) and total variation (TV) regularization of the solution (Viganò *et al.*, 2014 ▸). Under full-field illumination, grain maps can be generated up to about 2% total strain.

Reconstructions were managed with the DCT code hosted at the ESRF. This required a change in file format and transfer of the data during the experiment.

In addition the code was updated to take into account the specifics of the acquisition chain of the Psiché beamline. In parallel, efforts are ongoing to convert the current code to Python while accelerating reconstruction speed and improving user experience in a *Jupyter Lab* environment. Currently the pre-processing and segmentation steps have been implemented at Psiché. Resulting data can be input into the existing Matlab code to complete the DCT reconstruction. With this new pipeline, an acquisition consisting of 3600 images can be processed in less than 1 h (about 15 min for pre-processing and 30 min for segmentation) which can be up to one order of magnitude faster with respect to the current DCT code.

In order to ensure that DCT reconstructions are ready for mechanical simulations using finite element or FFT methods, additional numerical cleaning operations are performed with the Python *pymicro* package (https://github.com/heprom/pymicro). This includes morphological cleaning to eliminate small-artifact grains and final-grain dilation.

## 5. Results

In this section, the reconstructed data of DCT scans in the initial undeformed and subsequent deformed states for the same ROI are presented. Eventually both configurations are qualitatively mutually compared with EBSD measurements.

### 5.1. DCT reconstructions

**Figure 4:**
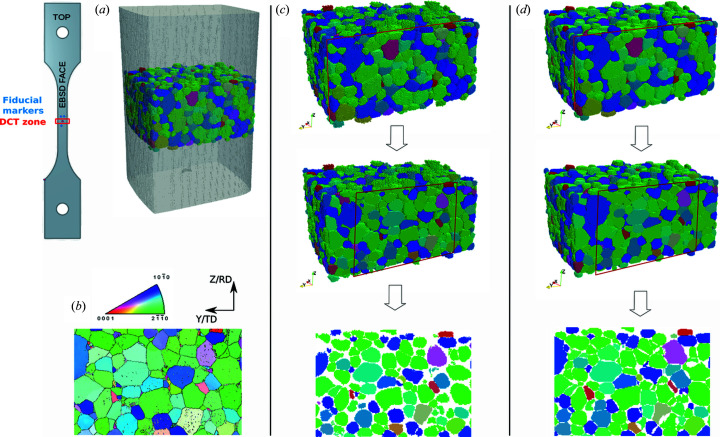
Visualization of DCT reconstructions in the undeformed configuration (IPF color coding along the *z* axis): (*a*) PCT reconstruction of the sample gauge length showing the DCT superimposed, (*b*) repolished EBSD map of the area corresponding to the DCT scan, (*c*) DCT SIRT reconstruction, (*d*) DCT 6DTV reconstruction; columns (*c*) and (*d*) show from top to bottom the full volume, the volume cropped as repolished and the slice corresponding to the EBSD map.

Raw DCT volume outputs from both SIRT and 6DTV reconstruction algorithms for the undeformed state are presented in Fig. 4 ▸ to assess the performance of the DCT reconstructions with the present setup at Psiché. Fig. 4 ▸ shows the results with the two algorithms side by side. Both algorithms reconstruct grains individually after the indexing phase leading to the same number of grains (1853). But not only does the 6D-DCT algorithm provide the full orientation field, it also improves the grain shapes significantly compared with EBSD. This is due to the fact that 6D-DCT accounts for local variations of the diffraction geometry. As a result it correlates more diffraction information which results in a more reliable grain shape. The expense is a more computationally intensive reconstruction: 10 h for SIRT and about 100 h for 6DTV [using 8 Intel Xeon cores with 256 Gb of RAM; note that for the 6DTV reconstruction the forward- and back-projection operations are handed over to the *Astra* toolbox (Palenstijn *et al.*, 2011 ▸) which runs on a Nvidia Titan X GPU card while the rest of the algorithm runs on a CPU]. For the remainder of this paper, the 6DTV reconstruction algorithm will be used.

**Figure 5:**
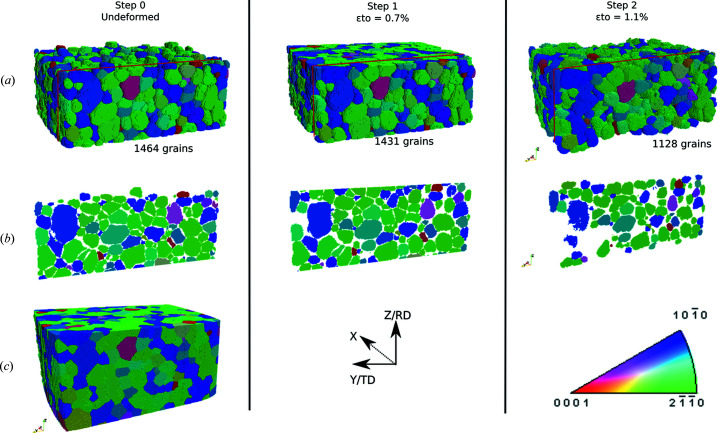
DCT reconstruction during *in situ* tensile test (IPF color coding along the tensile axis): (*a*) raw reconstructions of each loading step restricted to the common zone, (*b*) DCT slices corresponding to the repolished EBSD slice, (*c*) digital twin of the undeformed state after numerical cleaning and dilation.

Fig. 5 ▸ shows the DCT reconstructions at each step of the tensile test. As deformation proceeds, some grains are no longer indexed due to excessive overlap between diffraction spots. At step 1, 98% of the grains compared with the undeformed configuration can be reconstructed. At step 2, this reduces to 77%. In addition, the shape of reconstructed grains degrades as deformation proceeds, so that we lose more data at grain boundaries. For a more precise comparison of the performance of the reconstructions, we display slices corresponding to the re-polished EBSD surface, which have been extracted from the DCT volume data [see Fig. 5 ▸(*b*)].

The 6DTV microstructure was numerically dilated to generate the final volume [Fig. 5 ▸(*c*), step 0]. Note that only the undeformed state needs to be numerically dilated since it will be used later for crystal plasticity simulations (not discussed in this paper).

**Figure 6:**
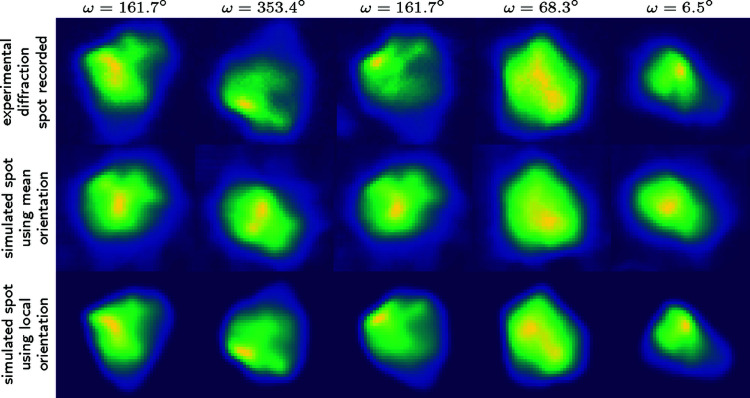
Illustration of the improved reconstruction quality using the local orientation field: experimental spots recorded on the detector for grain 44 are compared with simulated spots from the SIRT reconstruction (second row) and from the 6DTV reconstruction (third row), all images are plotted using arbitrary units and the same scale.

**Figure 7:**
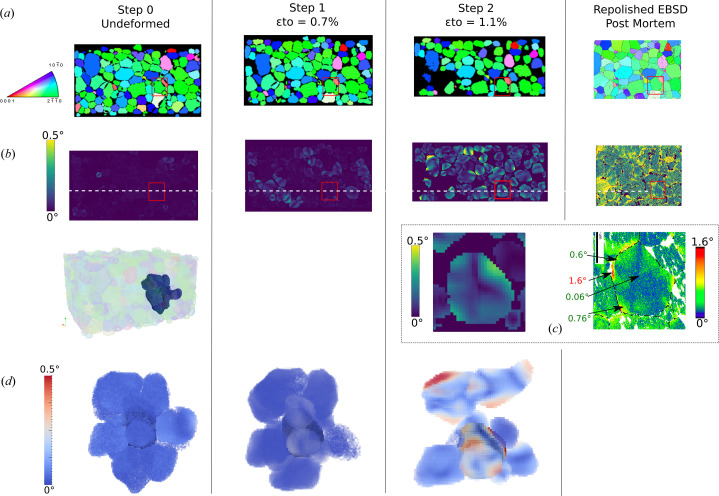
Study and validation of the GROD field from DCT. (*a*) IPF-Z DCT slices and corresponding repolished EBSD face, (*b*) GROD field slices evolution with load, (*c*) quantitative comparison between DCT and EBSD fields in the final deformation state, (*d*) 3D Paraview visualization of GROD field evolution for a grain and its environment.

Fig. 6 ▸ provides a further demonstration of the improved performance of the 6DTV algorithm in the case of a deformed microstructure. A forward simulation of the reconstructed data has been performed with a post-processing module available in the ESRF program in order to generate virtual spots which are compared with the experimental acquisition (first row in Fig. 6 ▸). The comparison with forward-simulated diffraction spots calculated for a constant (grain average) orientation in the grain shows that we lose much of the correspondence with experimental data (middle row). Indeed, the intensity distributions in the diffraction spots encode both grain shape and the local orientation field. As deformation proceeds, the information of the grain shape becomes progressively convoluted with the orientation field which distorts the spots. On the other hand, we notice that, when taking into account the local orientations resulting from the 6DTV reconstruction, the intensity distributions in the spots are closer to those observed experimentally than those from the 3D reconstruction. This captures the main trends of the real orientation field. Of course this does not prevent a direct comparison with another modality such as EBSD as shown later (see Fig. 7 ▸).

### 5.2. DCT analysis

#### 5.2.1. Grain reference orientation deviation fields

As the orientation field is directly available from the 6DTV reconstruction (in the form of a Rodrigues vector for each voxel), the grain reference orientation deviation (GROD) field was computed with respect to the average orientation in each grain for each load step.

Fig. 7 ▸ displays such fields for each load step in the slice corresponding to the re-polished EBSD face. As we deal with volume data, it is also possible to generate 3D visualizations of the GROD field for selected grains and their neighborhood [see Fig. 7 ▸(*d*)]. Qualitatively, we notice that, initially, the misorientation in each grain is negligible, confirming that the microstructure can be considered deformation-free in the reference state. In addition, a consistent evolution of the GROD field with loading is observed: in the intermediate load state (ɛ = 0.7%) the field is heterogeneous with clusters of higher activity and it homogenizes in most grains at the final load state (ɛ = 1.1%).

The DCT data were quantitatively validated by comparison with the GROD field in the re-polished EBSD on a few grains of interest. The activity is overall on the same order of magnitude in most of the grains. Also patterns are visually similar. However, we observe that, in the present case, DCT is not able to reconstruct close to the grain boundary where most of the misorientation takes place, especially close to triple junctions.

#### 5.2.2. Statistical analysis

**Figure 8:**
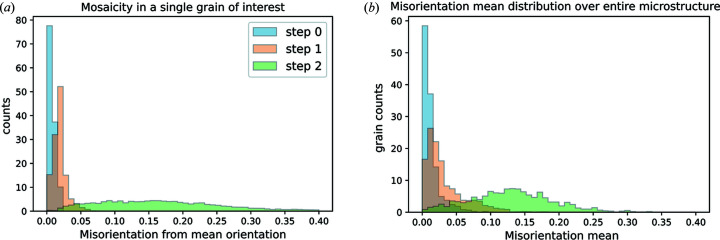
Statistical data extracted from the orientation data: (*a*) evolution of mosaicity on an entire grain of interest and (*b*) misorientation mean distribution evolution over the entire microstructure.

In addition to visualization of the deformation field, statistical data analysis can be carried out to plot the mosaicity evolution in a given grain [Fig. 8 ▸(*a*)] or the mean misorientation distribution evolution in the entire microstructure [Fig. 8 ▸(*b*)]. In agreement with the visual GROD observations, these quantities vary qualitatively as expected and can be used directly to compare with full-field crystal plasticity simulations.

## 6. Discussion

We observed that DCT acquisition in the undeformed state reconstructed with the 6DTV algorithm is able to reconstruct a reliable 3D grain map which compares well with the EBSD measurement in the bulk. We also saw that the grain shape obtained is not perfect near grain boundaries. In the present case, this is mainly attributed to the thickness of the scintillator selected at Psiché for the present experiment (50 µm thickness). Because the diffracted beams are not incident perpendicular on the scintillator, the extra thickness resulted in slightly blurred spots which impedes the ability of the detector to resolve the exact spot shapes. Also a few small grains are missed in the reconstruction process due to the selected segmentation thresholds. Indeed, with a large box beam acquisition, a compromise needs to be found between separation of diffraction spots and the precision of the segmentation. However, since the number of missing grains in the undeformed configuration is very limited and involves only the smallest grains, this is assumed to have negligible influence on the representativity of the microstructure. As a result, after dilation within the absorption mask, the grain map can be considered to be a reliable digital twin and used as input for subsequent full-field simulations.

Regarding the reconstruction of deformed volumes, the majority of the grains (77%) are still reconstructed after 1% strain, but an increasing number of grains are lost as deformation proceeds. This is caused by the increase of orientation spread in the grains which leads to diffraction spot overlap. This diffraction spot overlap and the drop of diffraction signal at the periphery of a grain are detrimental to fine quantitative analysis of the plasticity close to the grain boundaries, where most of the lattice misorientation accumulates. This effect can be counter-balanced to some extent by reducing the height of the illuminated sample volume. In the present study, we used slit heights from 278 µm to 220 µm for the different deformation states, corresponding to 1800 and 1400 illuminated grains per acquisition, respectively.

On the other hand, the observed evolution of the mis­orientation field in the central region of the grains remains consistent and compares well with EBSD data. The orientation data from these regions may be used for qualitative analysis of plastic activity. The extended (box) beam illumination used in DCT is the fastest technique to map thousands of grains, with isotropic spatial resolution in three dimensions. This is a major advantage when trying to produce a statistically representative analysis of microstructural events, especially as it gives direct non-destructive access to volume information of entire neighborhoods of grains.

**Figure 9:**
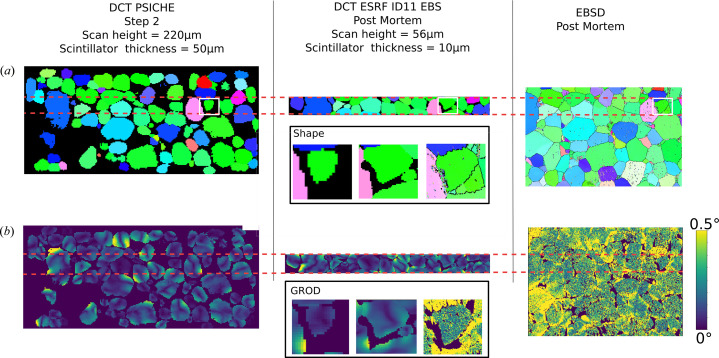
Comparison of the DCT performance between Psiché acquisition and the reference ESRF ID11 in the deformed state: (*a*) IPF-Z view of registered slices with EBSD, (*b*) GROD fields for each acquisition; insets show a zoomed-in view of a grain of interest.

In addition, as mentioned in Section 2, a *post mortem* DCT scan used as ground truth for the Psiché data validation has been performed at the ESRF ID11 beamline in a sub-region with optimized acquisition conditions for performance comparison [a DOI has been assigned to the raw data in the ESRF repository: https://data.esrf.fr/doi/10.15151/ESRF-ES-645553634 (Joste *et al.*, 2025 ▸)]. Using a scan height of 60 µm and a high-resolution detector system (10 µm free-standing LuAG screen), the user can significantly improve the accuracy of the grain shape and orientation field reconstruction (Fig. 9 ▸). We emphasize that the further degradation of the diffraction signal at higher levels of deformation can be handled using slice beam illumination and a forward modeling strategy (Suter *et al.*, 2006 ▸).

Regarding the GROD analysis and more specifically the comparison between DCT and EBSD data, many reasons can be invoked to explain the observed differences. Generally speaking, 6D-DCT is a mathematical optimization technique, trying to solve an under-determined, inverse problem using regularization techniques. This makes it inherently challenging (with respect to scanning techniques) to achieve similar accurate values. Especially close to grain boundaries (regions of intense modifications: discontinuities, dislocation accumulation, precipitates, defects), the diffraction signal is diffuse and less intense (limited volume). In other words, the signal-to-noise ratio is locally degraded. As a result this signal contribution is less likely to be taken into account. Moreover, the 6D-DCT framework only considers changes in crystal orientation and neglects elastic distortion of the crystal lattice. More advanced reconstruction techniques considering these additional degrees of freedom are still under development (Shen *et al.*, 2020 ▸; Reischig & Ludwig, 2020 ▸). For the present experiment, the thickness of the scintillator limits our ability to capture information close to grain boundaries. A thinner scintillator would improve the acquisition but at the cost of a longer scan time or a reduced signal-to-noise ratio. In the end, this is a parameter that can be adjusted within the available hardware to tune the compromise of precision versus acquisition speed. In addition, the level of deformation is also clearly a limiting factor. As mentioned, reducing the quantity of grains in the field of view allows reconstruction at larger deformation simply by decreasing the chance of spot overlap. The material choice can affect the performance when presenting a particular texture, annealing twins or precipitates at grain boundaries. Hence, all these reasons may yield non-reconstructed regions in the final volume. Numerical dilation based on orientation similarity is usually carried out to suppress them, particularly when material simulations are needed. In the present case, one may introduce a simple precision metric describing how well the DCT is able to capture the microstructure, using the ratio of voxels assigned to a grain over the total number of voxels in the illuminated part of the specimen. Using this metric, we found 81% in the initial configuration (step 0), 79% for step 1 and this value drops to 53% for step 2.

Meanwhile, the ff-3DXRD data are expected to bring additional information on local deformation mechanisms. For instance it can be processed using the *ImageD11* software (https://github.com/FABLE-3DXRD/ImageD11) which allows the user to index the grains (crystal mean orientation) within the illuminated volume. Further refinement of the diffraction peak positions allows the measurement of the mean elastic strain tensor in each grain. The average stress tensor for each grain can then be obtained using linear elasticity and the elastic constants of the material. The mean values obtained can be compared with simulation results and will be presented in another paper. This will allow us to take full advantage of the multimodal nature of the present experiment which facilitates the collection of richer datasets concurrently, as done on beamline 1-ID at the APS (Lienert *et al.*, 2011 ▸) or at CHESS (Nygren *et al.*, 2020*a* ▸). In the present paper, the experiment was conducted step by step but in principle it can be performed continuously as the Bulky stress rig allows movement of the cross head very slowly (50 nm s^−1^); this would require the acquisition of near-field and far-field modalities simultaneously as mentioned previously.

On other beamlines where the setup comprises a diffractometer, such as ID11 and ID06 at ESRF, other modalities become available during the experiment such as topo-tomography (Ludwig *et al.*, 2001 ▸, 2007 ▸; Proudhon *et al.*, 2018 ▸; Stinville *et al.*, 2022 ▸), scanning 3DXRD (Bonnin *et al.*, 2014 ▸; Hayashi *et al.*, 2019 ▸; Wright *et al.*, 2020 ▸; Henningsson *et al.*, 2020 ▸) or DFXM (Simons *et al.*, 2015 ▸). The ability to zoom in on a given grain or grain environment to observe at higher resolution will be key in future work to elucidate plasticity and fracture mechanisms based on a quantitative analysis of slip bands, geometrically necessary dislocation densities and intragranular elastic strain fields. Furthermore a crystal plasticity simulation (finite element or FFT) can be performed on real microstructures imaged by the DCT. Ultimately, the experimental data can be directly compared with the results of the simulations on a voxel-by-voxel basis (*i.e.* lattice orientation, elastic strain tensor). Overall, this approach represents a promising route towards the improvement of mechanical modeling.

## 7. Conclusions

We have demonstrated the feasibility of an *in situ* multimodal X-ray experimental setup on the Psiché beamline at Soleil applied to the study of polycrystal plasticity on a commercially pure titanium grade 2 material. This experimental apparatus enables the acquisition of DCT, PCT and ff-3DRXD modalities while keeping the sample installed on the test rig during a tensile test. We have successfully reconstructed grain maps from the DCT images at every load step by taking advantage of the DCT reconstruction code developed at the ESRF. The local rotation field obtained has been compared with *post mortem* EBSD measurements and showed good agreement. Despite a number of limitations in grains shape accuracy, the resulting digital microstructures allow consistent qualitative and statistical analysis of the orientation spread evolution in each grain as deformation proceeds. As an immediate benefit, this work demonstrates the possible use of a combination of near-field (DCT) and far-field (3DXRD) modalities for the user community at the SOLEIL synchrotron beamline.
